# MiR-744-5p inducing cell death by directly targeting HNRNPC and NFIX in ovarian cancer cells

**DOI:** 10.1038/s41598-018-27438-6

**Published:** 2018-06-13

**Authors:** Michael Kleemann, Helga Schneider, Kristian Unger, Philip Sander, E. Marion Schneider, Pamela Fischer-Posovszky, René Handrick, Kerstin Otte

**Affiliations:** 1 Institute of Applied Biotechnology, University of Applied Sciences Biberach, Hubertus-Liebrecht-Str. 35, 88400 Biberach, Germany; 2 University of Ulm, Faculty of Medicine, Albert-Einstein-Allee 11, 89079 Ulm, Germany; 3 Research Unit Radiation Cytogenetics, Helmholtz Center Munich, German Research Center for Environmental Health, Ingolstädter Landstr. 1, 85764 Neuherberg, Germany; 4 University Medical Center Ulm, Division of Experimental Anesthesiology, Albert-Einstein-Allee 23, 89081 Ulm, Germany; 5 University Medical Center Ulm, Division of Pediatric Endocrinology and Diabetes, Department of Pediatrics and Adolescent Medicine, Eythstr. 24, 89075 Ulm, Germany

## Abstract

MicroRNAs (miRNAs) play an important role in the regulation of gene expression. The binding to target messenger RNAs (mRNAs) results in mRNA cleavage or inhibition of the translational machinery leading to decreased protein levels. Various signalling pathways, including apoptosis are modulated by miRNAs. Here, we investigated the role of miR-744-5p in apoptosis signalling in ovarian cancer cell lines. MiR-744-5p expression was reduced in the cancer cell lines independent of the host gene MAP2K4. Overexpression of miR-744-5p activated the intrinsic apoptotic pathway in SKOV3, OVCAR3 and Cisplatin resistant (A2780-cis) and non-resistant A2780 cells leading to cell death. Notably, miR-744-5p overexpression together with Carboplatin treatment led to at least additive pro-apoptotic effects. Investigation of the apoptotic signalling pathways mediated by miR-744-5p revealed that its elevated expression directly downregulated mRNA and protein expression of nuclear factor I X (NFIX) and heterogeneous nuclear ribonucleoprotein C (HNRNPC). HNRNPC caused diminished miR-21 expression and AKT phosphorylation, while NFIX decreased Bcl2 levels, leading to the detected pro-apoptotic effects. Finally, Kaplan-Meier-Plots showed a prolonged median disease-free survival in ovarian serous cystadenocarcinoma patients with high miR-744 expression.

## Introduction

Apoptosis is a form of programmed cell death considered to destroy only single cells without damaging surrounding tissues^1^. It is induced via the interrelating and tightly controlled intrinsic and extrinsic apoptotic signalling pathways^1^. The extrinsic pathway is initiated by external signals as for example via the transmembrane receptor tumour necrosis factor receptor (TNFR)^1^, while the intrinsic pathway is induced by the release of mitochondrial cytochrome C. The integrity of mitochondria is mediated by different pro- and anti-apoptotic B-cell lymphoma 2 (Bcl2) members^2^. Bcl2-associated X protein (BAX), one of the pro-apoptotic members of this protein family induces apoptosis by the regulation of cytochrome C release from the mitochondria via alteration of mitochondrial membrane permeability^1^. Apoptotic signalling pathways are activating caspases^2^. Here the initiator caspase 8 is activated via extrinsic, caspase 9 more via intrinsic apoptosis pathway. Both caspases are activating the effector caspases 3 and -7^1^ and thereby finally leading to the cleavage of genomic DNA by caspase-activated deoxyribonucleases^3^ and cell shrinkage^4^. Apoptotic cells are eliminated via phagocytosis^1^.

MicroRNAs (miRNAs), around 22 nucleotides long, are single-stranded RNAs^5^. They are involved in the regulation of cellular processes such as apoptosis, proliferation or differentiation^6^. Due to the fine tuning of the apoptosis regulation^7^ and the increasing evidence as potential tumour suppressor genes, miRNAs are highly interesting molecules for the generation of novel anticancer therapeutics^8^.

MiRNAs are transcribed by RNA-polymerase II and processed by the enzymes drosha ribonuclease III (DROSHA) and dicer 1 ribonuclease III (DICER). The miRNAs are bound by Argonaut proteins (AGO2) to the RNA induced silencing complex (RISC). RISC binds to the 3 prime untranslated region (3′UTR) of a target gene and thereby functions as post-transcriptional regulator^9^. The binding of a miRNA to the target mRNA typically leads to translational repression and mRNA decay, although highly complementary targets can be cleaved endonucleolyticaly^9^. MiRNAs bind with imperfect base pairing to their targets of multiple genes, and can therefore interact with several signalling pathways^10^.

MiRNA-744 is known to be significantly deregulated in several cancers, for example in human hepatocellular carcinoma, pancreatic, colon or gastric cancer^11^, leading to its investigation as a prognostic biomarker in hepatocellular carcinoma and pancreatic cancer^12,13^. Due to its deregulation miR-744 has been hypothesized to play an important role in tumour development or tumourigenesis^11^. However, its role in ovarian cancer and the underlying mechanisms leading to the observed cellular responses are unknown. Ovarian cancer (ovarian CA) is a common human cancer with poor prognosis and the highest death-to-incidence ratio^14^. It refers to a highly heterogeneous tumour type including the subgroup of epithelial ovarian carcinoma^15^. Early detection of ovarian CA is very difficult and limited by the method spectra^16^. For cancer therapy, researcher focus on oncogenes, tumour suppressors as well as cell signalling pathways exploring their role in tumour progression by inducing proliferation or inhibition of apoptosis^17^.

Based on a previous high throughput screening analysing 188 miRNAs in different cancer cell lines^18^ we identified several novel miRNAs to induce cell death in ovarian CA cell lines. The aim of this study was to identify the role of miR-744-5p in programmed cell death of ovarian CA cell lines and analyse underlying cellular mechanisms by identifying target genes regulated by miR-744-5p involved in signalling pathways leading to the cellular response of cell death.

## Results

### MiR-744-5p, a pro-survival miRNA in patients with ovarian carcinoma

**Figure 1:**
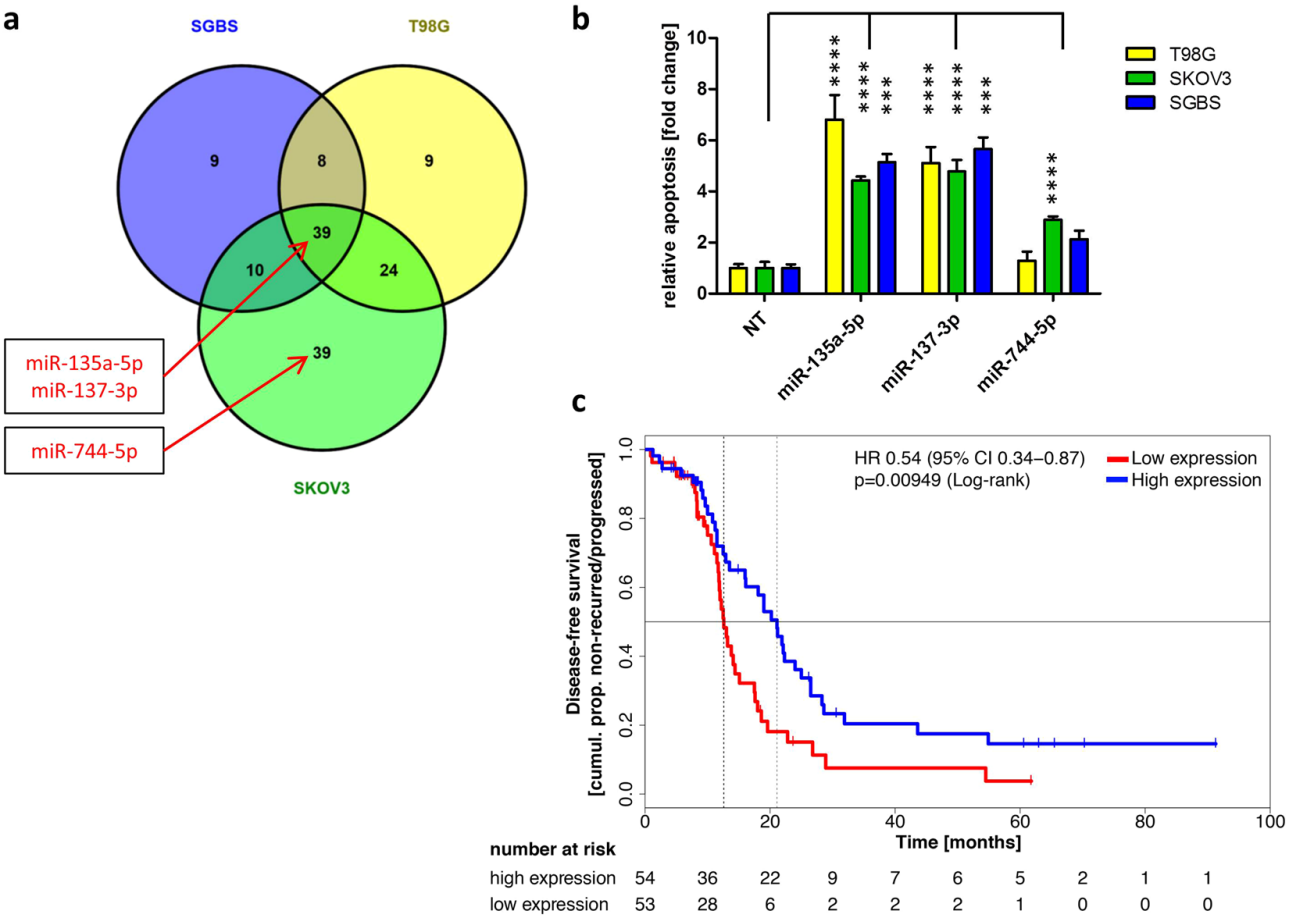
MiRNA screen validation and Kaplan-Meier-Plot for miR-744. MiRNAs with significant (fold change higher 1.5 and p < 0.01) pro-apoptotic effects were clustered in a Venn diagram (**a**). For validation screening T98G, SKOV3, and SGBS cells were seeded 24 h before transfection with miRNA mimics (50 nM and 0.4 µl ScreenFect A) or non-targeting siRNA (NT, negative control for cell death) control. Apoptosis rates 72 h after transfection were analysed by Nicoletti staining followed by flow cytometric analysis. Data were normalized to NT control of the referring cell line (**b**). Kaplan-Meier analysis of elderly (cohort older than 67.5 years) ovarian cancer patients with high (blue) and low expression (red) of hsa-miR-744. The median expression of the miRNA was used as a cut-off criteria for patient dichotomization. Disease-free survival (DFS) was used as endpoint with recurrence or progression defining an event. High and low expressors exhibited a median disease-free survival of 19 and 14.1 months, respectively (**c**). Statistical analyses were performed by one-way ANOVA followed by Bonferroni post-test. The effect of the transfected miRNAs were compared to NT [n = 3 biological replicates; mean ± SD; ***p < 0.001; ****p < 0.0001].

Due to their involvement in the regulation of fundamental cellular processes such as apoptosis, miRNAs are promising candidate molecules for the generation of novel anticancer therapeutics. Therefore, we previously performed an apoptosis screening employing 188 miRNAs in various human cancer cell lines including an ovarian carcinoma (SKOV3) and a glioblastoma (T98G) cell line^18^. In addition, SGBS preadipocyte cells were employed as a non-immortalized and non-cancerous control cell line^19^. Apoptosis induction by miRNAs were accessed by transient miRNA mimics transfection and subsequent apoptosis measurement by quantitative flow cytometry. To identify miRNAs with cell line specific or overlapping functions, we clustered miRNAs inducing apoptosis compared to a non-targeting control (NT) with a fold change of greater 1.5 and a p-value lower 0.01 (Fig. 1a). Here, miR-744-5p induced apoptosis only significantly (p < 0.0001) in SKOV3 cells with a fold change of 2.89 fold +/− 0.13 fold at 72 h after transfection compared to a non-targeting control (NT, 1.00 fold +/− 0.24 fold), but no significant induction of apoptosis was observed for T89G or SGBS cells (Fig. 1a,b). The previously described pro-apoptotic miRNAs miR-135a-5p^20,21^ and miR-137-3p^22^ were identified as positive controls in all three cell lines. Here, miR-135a-5p induced apoptosis rates of 4.43 fold +/− 0.15 fold in SKOV3 cells compared to 6.80 fold +/− 0.97 fold in T98G cells (Fig. 1b).

To further assess the potential of miR-744 as a diagnostic or therapeutic agent, data from ovarian CA patients from The Cancer Genome Atlas (TCGA) were analysed and revealed a hitherto unknown pro-survival function of miR-744 in these patients. The median of miRNA expression was used as cut-off criteria for the Kaplan-Meier-Plots. The disease-free survival (DFS) was used as endpoint. We showed that older female patients (older than 67.5 years) with a high expression of hsa-miR-744 have a 5.1 months prolonged median DFS (hazard ratio (HR) 0.54, p 0.009) compared to patients with a low expression of hsa-miR-744 (19.0 months vs 14.1 months) pointing toward a potential protective effect of miR-744 (Fig. 1c).

### Altered miR-744 basal expression level in ovarian cell lines and its modulation during apoptosis induction

**Figure 2:**
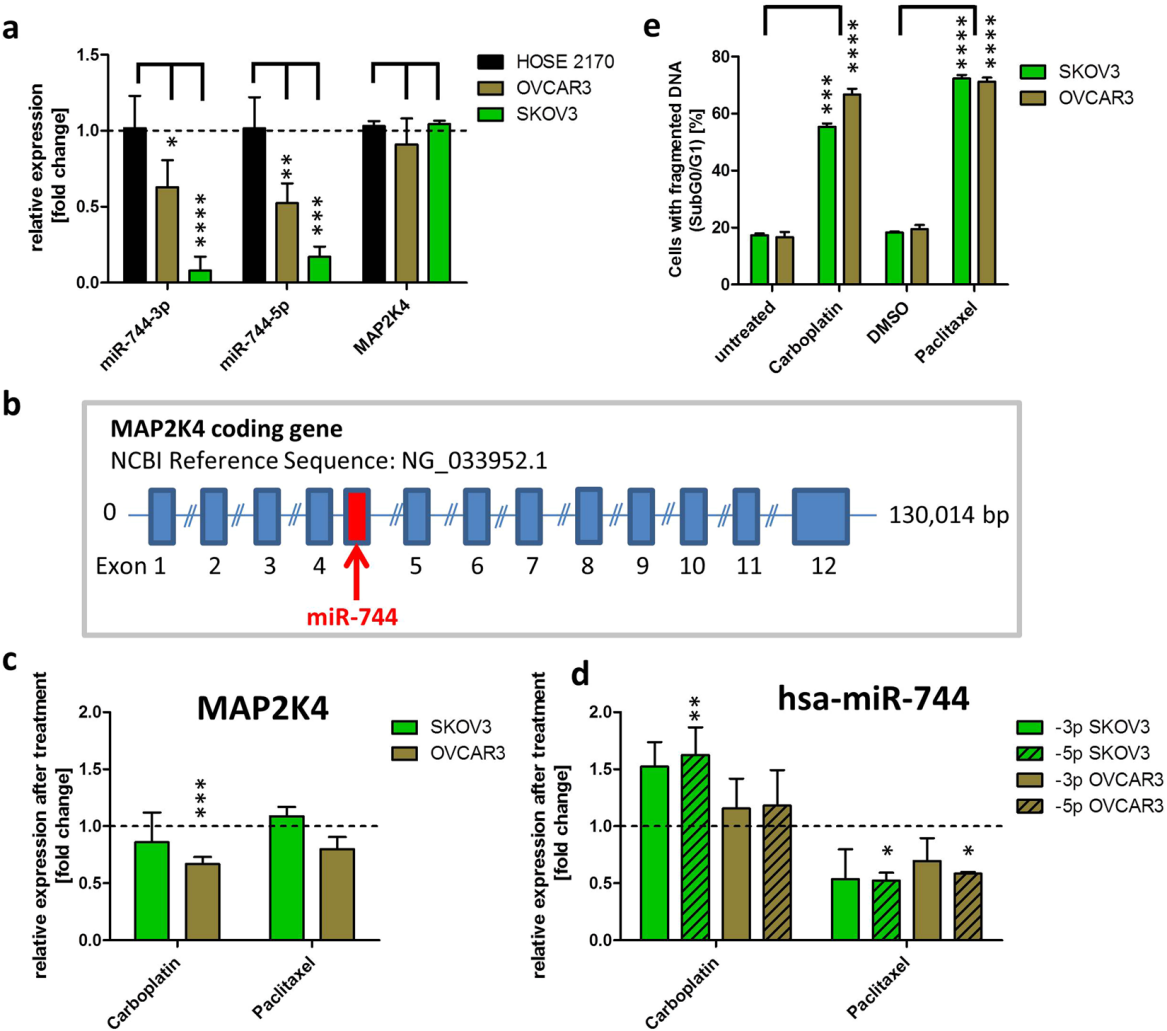
Expression of hsa-miR-744 and its host gene MAP2K4. qRT-PCR of untreated HOSE 2170, SKOV3 and OVCAR3 cells for miR-744-3p and -5p expression as well as the expression of MAP2K4 (**a**), the host gene of the miRNA (schematic view, **b**). In order to determine MAP2K4 (**c**) and miR-744 expression in SKOV3 and OVCAR3 cells (**d**) after induction of apoptosis (Nicoletti assay 48 h after treatment - **e**), cells were seeded 24 h prior treatment with 80 µM Carboplatin or 0.25 µM Paclitaxel for additional 48 h. The expression analysis of miR-744 was normalized to the CT value of U6 snRNA and the expression of MAP2K4 was normalized to the CT value of PPIA^55^. Statistical differences were tested using paired t-test [n = 3 biological and technical replicates; mean ± SD; ^*^p < 0.05; ^**^p < 0.01; ^***^p < 0.001; ^****^p < 0.0001].

Since the expression of miRNAs in cancer cells was reported to be often deregulated^23^, the expression of both miR-744 strands in the ovarian CA cell lines SKOV3 and OVCAR3 were compared to the non-cancerous ovarian cell line human ovarian surface epithelial (HOSE) 2170 derived from healthy tissue^24^. The expression of hsa-miR-744-5p in ovarian CA cells was diminished to 0.52 fold +/− 0.13 fold in OVCAR3 cells and even more strikingly to 0.17 fold +/− 0.07 fold in SKOV3 cells compared to the non-cancerous HOSE cells (Fig. 2a).

MiR-744 is encoded on the human chromosome 17p12 in the coding region for mitogen-activated protein kinase 4 (MAP2K4), placed in intron 4 in close proximity to exon 4 (Fig. 2b). To ensure that the expression of MAP2K4 is not altered in cancer cells qRT-PCR analysis was performed. In contrast to the intronic miR-744, expression levels of the miRNA host gene were not reduced in cancer cell lines. As shown in Fig. 2a, almost equal mRNA levels were present in both cancer cell lines, OVCAR3 and SKOV3, as well as in the normal cell line HOSE 2170.

To assess whether chemotherapeutics influence miR-744 expression non-treated cells were compared with those treated with Carboplatin and Paclitaxel. For ovarian cancer patients chemotherapy most commonly employs Carboplatin and Paclitaxel treatment for 3 to 6 cycles^25^. Paclitaxel inhibits the cell division by binding to α-tubulin and thereby stabilizing the microtubules^26^. The activation of apoptosis by taxol treatment is caspase 3 and −9 independent^27^. Carboplatin leads to apoptosis induction by DNA cross linking^28^. To analyse the expression of MAP2K4 after apoptosis the cells were treated with 80 µM Carboplatin or 0.25 µM Paclitaxel for 48 h. This led to an apoptosis rate of 72.29% +/− 1.27% in SKOV3 and 71.21% +/− 1.40% in OVCAR3 cells (Fig. 2e). Analysis of the MAP2K4 expression revealed a downregulation in OVCAR3 cells. Here, the mRNA levels were significantly reduced to 0.66 fold +/− 0.06 fold (p < 0.001) after treatment with Carboplatin while treatment with Paclitaxel only moderately altered MAP2K4 expression in SKOV3 and OVCAR3 cells (Fig. 2c). However, upon Carboplatin induced apoptosis, the expression of miR-744-5p was significantly (p < 0.01) enhanced to 1.62 fold +/− 0.24 fold compared to untreated SKOV3 cells. Paclitaxel mediated reduction of hsa-miR-744-5p expression was almost comparable in both cell lines (reduced to 0.52 fold +/− 0.07 fold in SKOV3 cells and to 0.58 fold +/− 0.02 fold in OVCAR3 cells, Fig. 2d), potentially indicating different expression and regulation mechanisms for miR-744 and its host gene MAP2K4.

### MiR-744 induces cell death in SKOV3 cells

**Figure 3:**
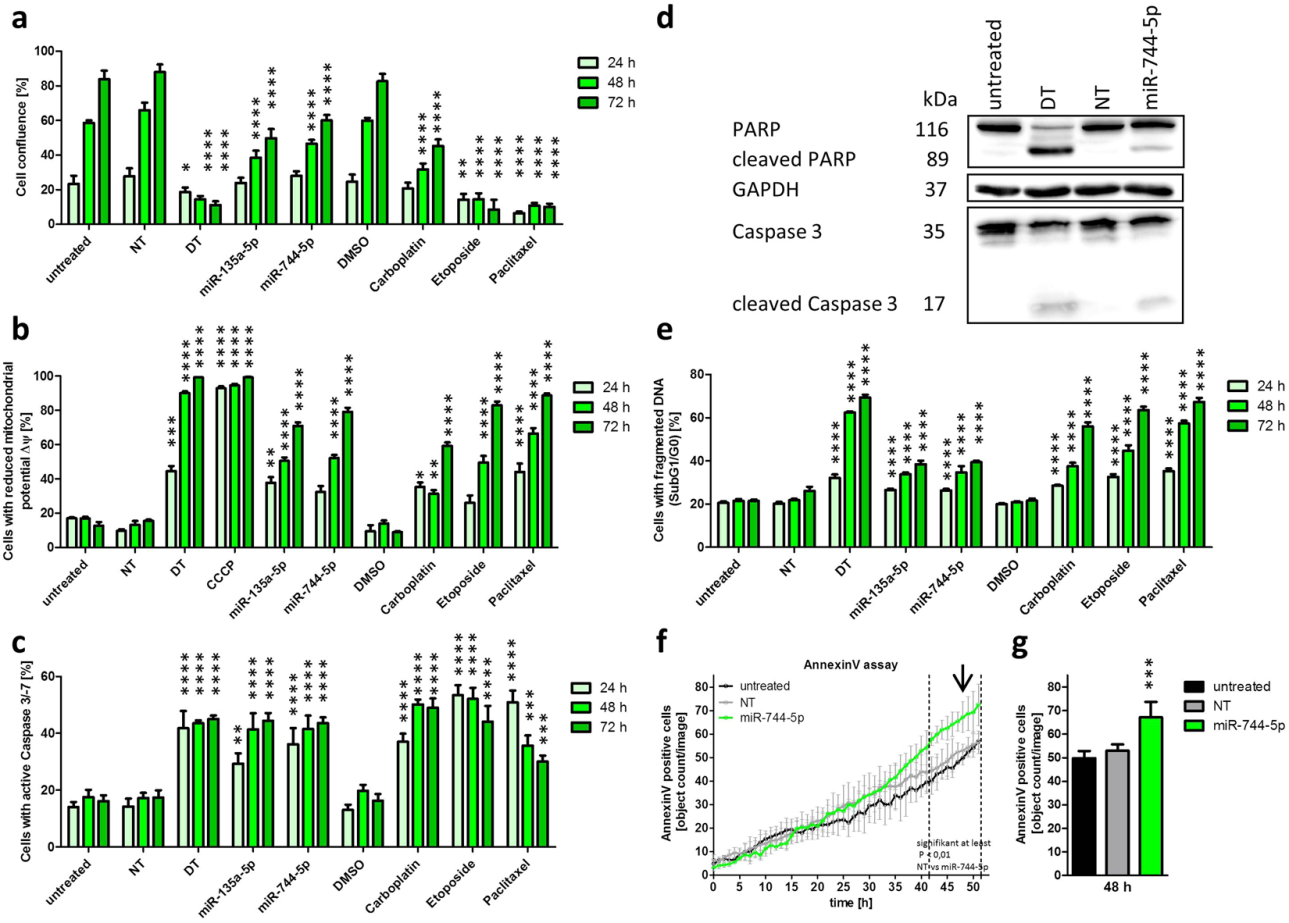
Time course and hallmarks of miR-744 induced apoptosis. SKOV3 cells were seeded into 96 well plates 24 h prior transfection with 62.5 nM miR-744 mimics, non-targeting siRNA (NT, negative control for cell death), cell death inducing siRNA (DT, functional transfection control, positive control for cell death) or treatment with Caboplatin, Etoposide or Paclitaxel. 24 h, 48 h and 72 h after treatment the cells were analysed for their cell confluence (**a**), their loss of mitochondrial potential ∆Ψm (**b**), the activity of caspase 3 and −7 (**c**) and their content of fragmented DNA (cells in sub G0/G1, **e**). 5 µM CCCP served as a positive control for the breakdown of mitochondrial potential ∆Ψm (**b**). AnnexinV staining was performed by long term imaging using IncuCyte AnnexinV Red Reagent (**f**). Quantitative analysis of AnnexinV determined at 48 h (arrow) was significantly different for miR-744-5p compared to NT control (**g**). For Western Blot analysis (**d**) the cells were harvested 60 h after treatment. The blots were stained with antibodies against Caspase 3, cleaved Caspase 3, PARP and cleaved PARP. GAPDH was used as loading control. Statistical analysis were performed by one-way ANOVA followed by Bonferroni post-test (**a**,**b**,**c**,**e**) as well as two-way ANOVA analysing the AnnexinV measurement (f and g) [n = 3 biological replicates; mean ± SD; ^*^p < 0.05; ^**^p < 0.01; ^***^p < 0.001; ^****^p < 0.0001].

In order to investigate the pro-apoptotic potential of miR-744-5p, we applied several flow cytometric methods to assess molecular changes in apoptotic pathways. To this end, we chose SKOV3 cells since these cells have been investigated in a previous apoptosis screening (Fig. 1a and b). The amount of miR-744 was increased upon transient miR-744-5p mimic transfection into SKOV3 cells, which show low endogenous miR-744 expression. In these experiments, miR-135a-5p served as a positive control for pro-apoptotic effects induced by miRNAs. By using the NyONE microscope, analysis of cell confluence revealed that the apoptosis inducing siRNA death control (DT) significantly (p < 0.0001) reduced cell confluence over the whole time course to 11.2% +/− 2.1% after 72 h (Fig. 3a). The apoptosis inducing chemotherapeutic agents Etoposide and Paclitaxel diminished the cell confluence to 8.4% +/− 5.8% and 10.1% +/− 1.7% 72 h after treatment, respectively. Compared to the non-targeting control (NT), miR-744-5p reduced the cell confluency to 60.0% +/− 3.2% and control miR-135a-5p to 49.8% +/− 5.2%. As the intrinsic apoptotic pathway is activated by the release of cytochrome C the mitochondrial membrane potential ΔΨm is reduced^2^. Nearly all cells treated with the positive control carbonylcyanid-m-chlorphenylhydrazon (CCCP; 99.33% +/− 0.28%) showed a low ΔΨm after staining with Tetramethylrhodamine ethyl ester (TMRE). In about 90% of the DMSO treated cells, a normal mitochondrial ΔΨm was measured at the indicated time points. MiR-744 transfection led to a reduction of the ΔΨm in 79.01% +/− 2.47% of the cells, which was in a similar range induced by Etoposide (83.02% +/− 2.08%) or Paclitaxel (88.67% +/− 1.24%; Fig. 3b).

The activation of initiator caspases is described to start early in the apoptotic process^1^, although the activity of the effector caspase 3 and −7 was measured with the CellEvent Caspase-3/7 Green Flow Cytometry Assay Kit 24 h after transfection. At 48 h after transfection, miR-744 led to an activation of caspase 3 and −7 in 41.53% +/− 4.70% of the cells. Both, Etoposide and Paclitaxel increased the activity of caspase 3 and −7 already 24 h after treatment up to 53.45% +/− 3.52% and 50.90% +/− 4.16%, compared to basal levels with NT control (14.23% +/− 2.78%) respectively (Fig. 3c). To substantiate these results, Western Blot analysis was performed 60 h after transfection. This time point after transfection was determined in previous experiments to reveal the highest detectable molecular changes whereas at later time points the degradation in apoptotic cells was too high. A substantial increase in cleaved Poly (ADP-ribose) polymerase (PARP) as well as a cleavage of Caspase 3 in DT and miR-744 transfected cells was detected (Fig. 3d, densitometry Supplementary Fig. S1). During apoptosis caspase-activated DNase (CAD) cleaves the DNA^29^ resulting in short DNA fragments which can be measured as SubG0/G1 fraction by flow cytometry after Nicoletti staining. DT, Etoposide and Paclitaxel treated controls revealed the highest amount of fragmented DNA with up to 69.36% +/− 1.37% (DT) at 72 h. After miR-744-5p transfection the amount of fragmented DNA increased over time leading to a 1.5 fold increased DNA fragmentation (72 h after transfection) when compared to NT transfected cells (39.43% +/− 0.64% vs 26.18% +/− 1.87%, respectively; Fig. 3e). Next an AnnexinV staining was performed to verify apoptotic alterations in phosphatidylserine plasma membrane asymmetry of the cells. To avoid cell membrane destruction induced by later necrotic effects influencing the level of detectable AnnexinV, the assay was performed for 51 h. In SKOV3 cells transfected with miR-744-5p, the amount of AnnexinV positive cells was significantly higher when compared to NT transfected cells (from 41 h onwards, p < 0.05; two-way ANOVA; Fig. 3f,g).

### Combined effects of miR-744 transfection and Carboplatin treatment

**Figure 4:**
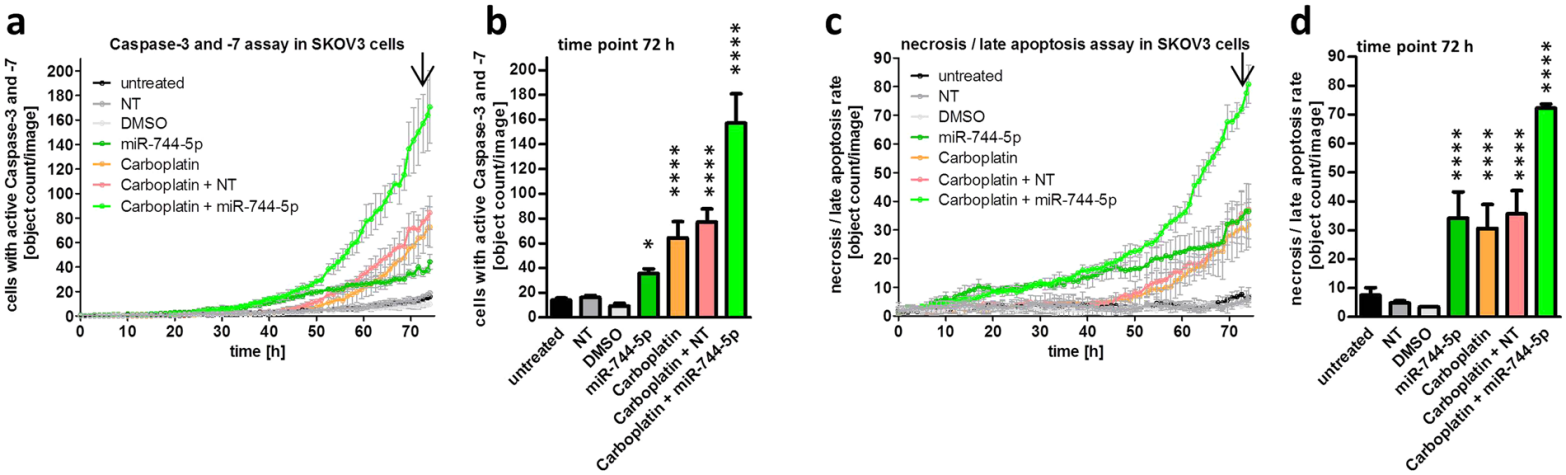
Combined effect of miR-744 with Carboplatin. For long term online analysis SKOV3 cells were seeded and transfected as described in Fig. 3. In order to detect apoptosis induction as well as late apoptosis and necrosis by miR-744 cells were stained with IncuCyte Caspase-3/7 Green Apoptosis Assay Reagent (**a**) or propidium iodide (**c**), respectively. The cells were automatically photographed every hour by the IncuCyte ZOOM System. The amount of stained cells was calculated by the IncuCyte ZOOM Software. Significance was proven using two-way ANOVA followed by Bonferroni post-test referred to NT control (negative control for cell death). Calculation was done for the time point 72 h (b and d) [n = 3 biological replicates; mean ± SD; ^*^p < 0.05; ^****^p < 0.0001].

**Figure 5:**
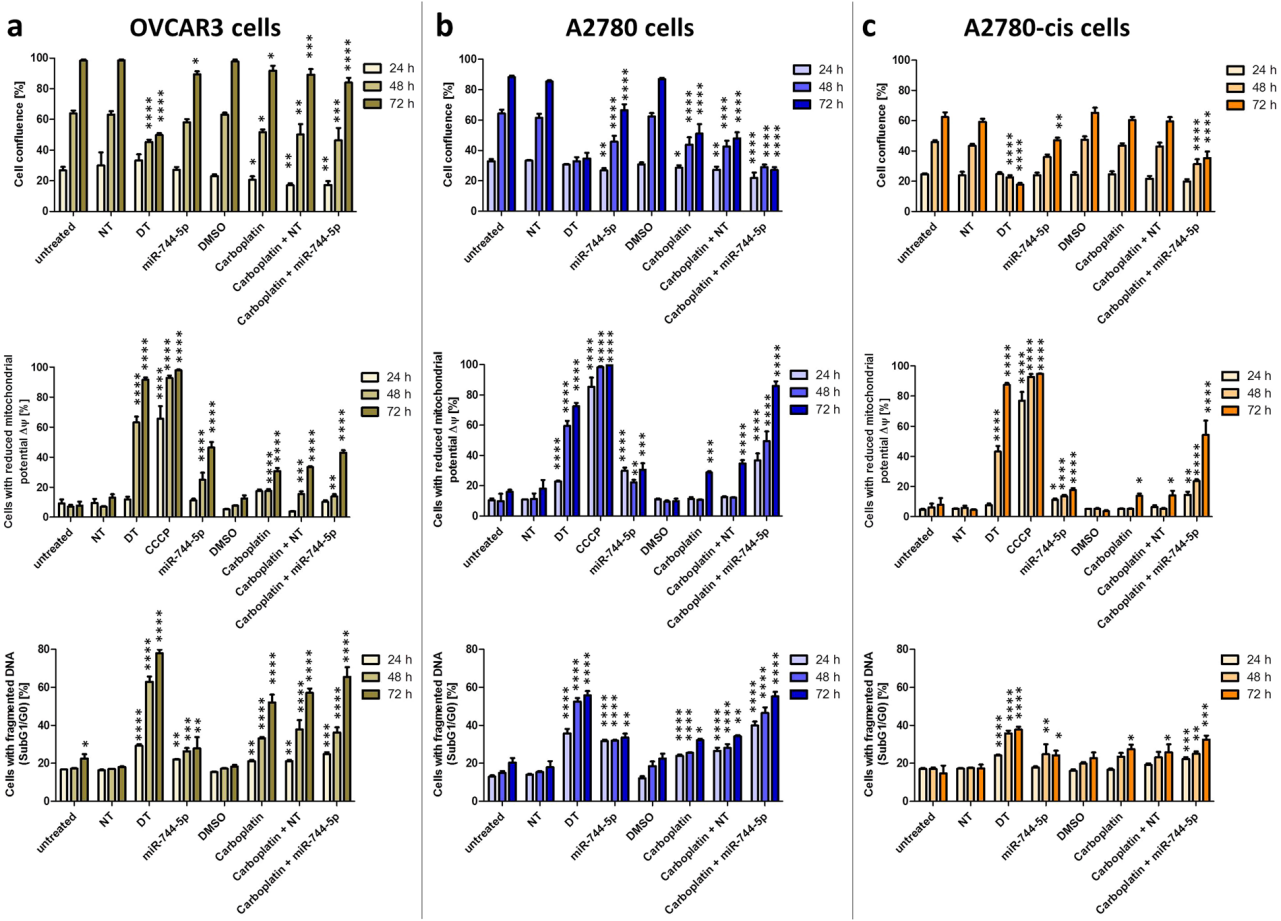
Pro-apoptotic effects of miR-744 in combination treatment of A2780, A2780-cis and OVCAR3 cells. For the validation of pro-apoptotic effects OVCAR3 (**a**), A2780 (**b**) and Cisplatin resistant A2780 (A2780-cis, **c**) cells were seeded and transfected as described in Fig. 3. A non-targeting siRNA (NT) served as a negative and a cell death inducing siRNA (DT) served as a positive control for cell death. In order to test the effect of Carboplatin also in combination with miR-744 the cells were treated with Carboplatin. 24 h, 48 h and 72 h after treatment the cells were analysed for their cell confluence (upper panel), their loss of mitochondrial potential ∆Ψm (middle panel) and their content of fragmented DNA (cells in sub G0/G1, lower panel). 5 µM CCCP served as a positive control for the breakdown of mitochondrial membrane potential ∆Ψm (middle panel). Statistical analysis was performed by one-way ANOVA followed by Bonferroni post-test [n = 3 biological replicates; mean ± SD; ^*^p < 0.05; ^**^p < 0.01; ^***^p < 0.001; ^****^p < 0.0001].

In order to analyse potential synergistic and sensitizing effects with a chemotherapeutic agent already in use for ovarian CA treatment^25^, Carboplatin was tested in combination with miR-744-5p in SKOV3 cells. For this, SKOV3 cells were seeded, transfected, treated with Carboplatin and analysed every hour by long-term video-microscopy using the IncuCyte ZOOM Live-Cell Analysis System. As shown in Fig. 4, Carboplatin treatment alone induced a moderate caspase 3 and −7 activation starting at approximately 45 h after treatment. Strikingly, already 25 h after treatment, cells transfected with miR-744-5p only as well as cells receiving combination treatment (Carboplatin and miR-744-5p) induced the activity of caspase 3 and −7. However, 45 h after treatment, the effect of Carboplatin increased and in combination with miR-744-5p, led to a far higher effect of 3.9 fold compared to miR-744-5p transfection only (Fig. 4a,b). A significant increase of p < 0.05 was analysed by two-way ANOVA followed by Bonferroni post-test. It showed for Carboplatin after 58 h, for Carboplatin + NT after 53 h, for hsa-miR-744-5p after 45 h and for Carboplatin + hsa-miR-744-5p after 41 h significant differences compared to untreated cells. This effect was also detected in the PI-exclusion assay measuring the induction of cell death by long-term video-microscopy. It started already 5 h after transfection and led after 75 h to a 2.2 fold increase in cells which received the combination treatment when compared to miR-744-5p alone including late apoptotic and necrotic cells (Fig. 4c,d, significant increase of p < 0.05 calculated by two-way ANOVA followed by Bonferroni post-test: Carboplatin after 59 h, Carboplatin + NT after 53 h, hsa-miR-744-5p after 25 h and Carboplatin + hsa-miR-744-5p after 27 h). The observed elevation in cell death showed at least additive effects for the combination treatment of Carboplatin and miR-744-5p. In order to substantiate the observations on pro-apoptotic effects of miR-744-5p alone and in combination treatment with Carboplatin, further ovarian cancer cell lines with different genetic background were investigated. These included besides SKOV3^p53null^ the highly invasive cell line OVCAR3^p53R248Q^ ^30^ as well as the cell lines A2780-cis^p53K351N^ and A2780 with and without carboplatin resistance, respectively^31,32^. OVCAR3 cells showed a time dependent increase in cell confluence over 72 h, which was reduced after transfection with the death control siRNA (DT), miR-744-5p and with the single and combined treatment with Carboplatin. The amount of cells with reduced mitochondrial potential was analysed by TMRE assay. In all three cell lines OVCAR3, A2780 and A2780-cis, the amount of cells with reduced mitochondrial membrane potential after treatment increased over time. In Cisplatin resistant A2780 cells, the combination treatment with Carboplatin and miR-744 lead to an increase by at least 54.20% +/− 9.49% 72 h after treatment. The amount of cells with fragmented DNA increased from 18.01% +/− 0.35% (NT control) to 27.85% +/− 5.91% (miR-744-5p transfected cells) and even 65.59% +/− 5.03% after combined treatment with Carboplatin and miR-744 (Fig. 5a). When A2780 cells (Fig. 5b) were compared to Cisplatin resistant A2780 cells (A2780-cis, Fig. 5c) a reduced activation of death signals was observed for the Cisplatin resistant cells, as the effects were always lower in A2780-cis than in A2780 cells. A significant growth inhibition in A2780-cis cells was only detectable after transfection with the death control siRNA (DT) or miR-744-5p as well as Carboplatin in combination with miR-744, which reduced A2780-cis cell growth and sensitized the cells for cell death. In the p53 wildtype A2780 cells miR-744 as well as the treatment with Carboplatin lead to significantly more cells with reduced mitochondrial membrane potential and with fragmented DNA. In all four cell lines investigated, the combination treatment of miR-744 together with Carboplatin caused the highest amount of apoptotic cells (Figs 4 and 5).

### NFIX and HNRNPC regulation by exogenous miR-744-5p

**Figure 6:**
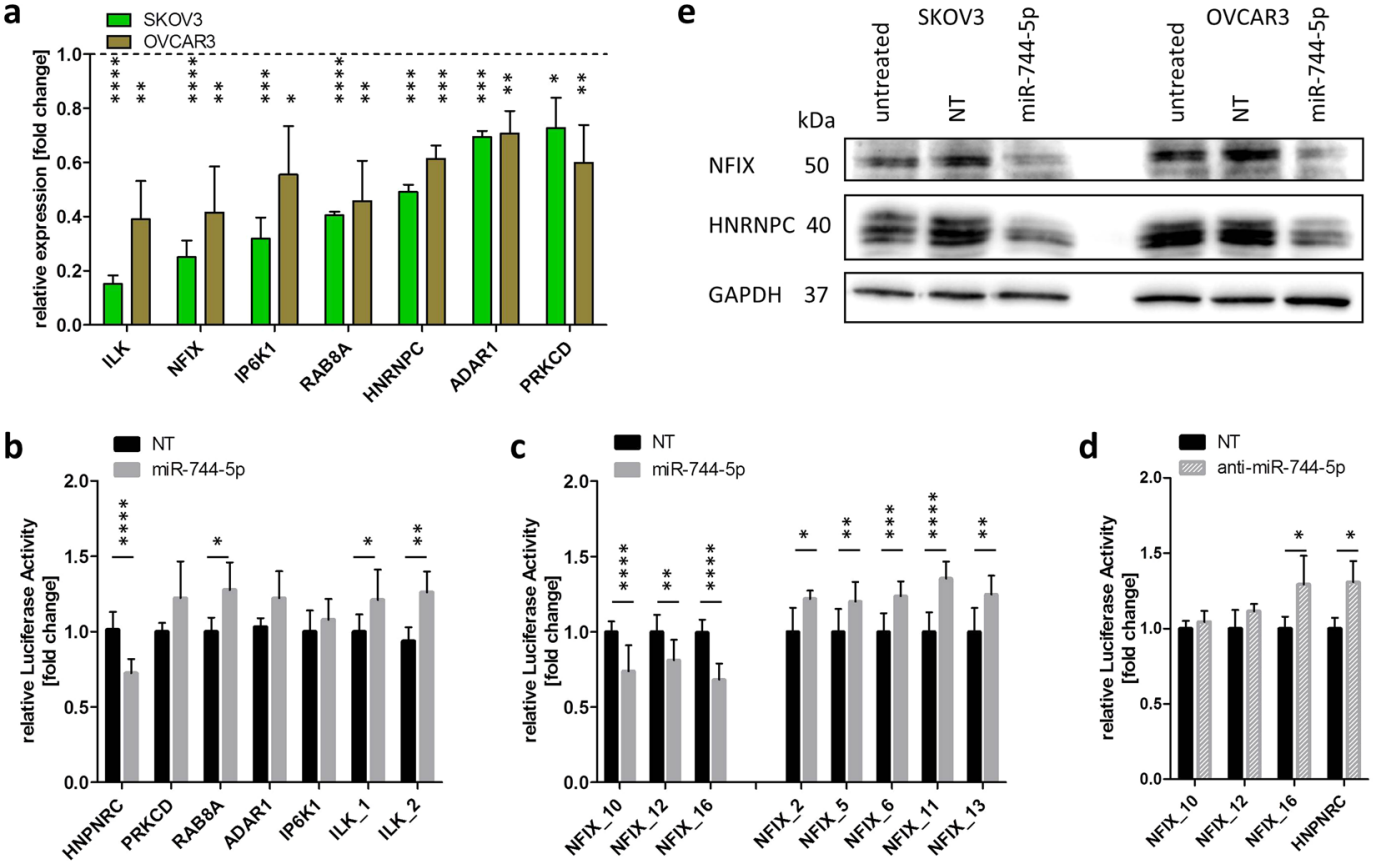
HNRNPC and NFIX as direct targets for miR-744. To validate miR-744 mediated regulation of potential target genes, SKOV3 and OVCAR3 cells were transfected with miR-744 mimic or non-targeting siRNA (NT, negative control for cell death) as described in Fig. 3. After 48 h, potential target gene expression was analysed by qRT-PCR. The relative mRNA expression of potential target genes was normalized to PPIA and NT employing the Livak method^55^ (**a**). Significantly downregulated potential targets were further analysed by the Luciferase Reporter Assay (**b** and **c**) to proof direct binding of the miRNA to its predicted target sequence. Anti-miR-744 was used to confirm the binding sides of miR-744 to the target mRNAs (**d**). The binding sites in the 3′UTR (Table 3), based on data of microRNA.org^34^ and TargetScanHuman^35^, were cloned into a pMirGLO vector. Relative luciferase activity was measured 72 h after co-transfection of the pMirGLO vector with miR-744 mimic, miRNA inhibitor anti-miR-744 or non-targeting siRNA (NT) in HEK293T cells. The relative luciferase expression was normalized to the expression after co-transfection with NT. For Western Blot analysis, SKOV3 and OVCAR3 cells were harvested 60 h after miR-744 transfection (**e**). The representative blots were stained with antibodies against NFIX and HNRNPC. GAPDH was used as loading control. Statistical differences were tested using paired t-test [n = 3 biological and technical replicates; mean ± SD; ^*^p < 0.05; ^**^p < 0.01; ^***^p < 0.001; ^****^p < 0.0001].

To analyse potential targets that might lead to the detected effects of cell death, TCGA data from the “Ovarian serous cystadenocarcinoma” data set were evaluated regarding negative correlation of miR-744-5p expression to the expression of potential target mRNAs or proteins. Here, a high miRNA expression level should correlate with a low level of target mRNA or protein. Potential targets with the lowest correlation coefficient (high correlation; cor. coef) and a p-value smaller 0.05 were processed further (for correlation based on mRNA see Table 1; correlation based on protein level is given in Table 2). The online gene classification software PANTHER (Protein Analysis Through Evolutionary Relationships)^33^ was used to cluster the potential targets for apoptotic functions. Potential targets with apoptotic function and predicted binding sides to miR-744 (based on the databases microRNA.org^34^ and TargetScanHuman^35^) were selected for quantitative real-time polymerase chain reaction (qRT-PCR) analysis in both SKOV3 and OVCAR3 cells. After transient transfection of miR-744-5p and subsequent qRT-PCR analysis, the potential targets integrin linked kinase (ILK, 2 binding sides), nuclear factor I X (NFIX, 16 binding sides), inositol hexakisphosphate kinase 1 (IP6K1), Ras-related protein Rab-8A (RAB8A), heterogeneous nuclear ribonucleoprotein C (HNRNPC), adenosine deaminase 1 (ADAR1) and protein kinase C delta (PRKCD) showed significantly (p < 0.05) reduced expression when compared to the NT control (Fig. 6a). Although revealing correlation coefficients with p-values smaller 0.05, CGG triplet repeat binding protein 1 (CGGBP1), cyclin dependent kinase inhibitor 2A (P16INKA4), eukaryotic translation initiation factor 4A2 (EIF4A2), zinc finger protein 653 (ZNF653) and family with sequence similarity 63 member A (FAM63A) were not detectable by qRT-PCR in SKOV3 and OVCAR3 cells (data not shown).

**Table 1:** miRNA correlation to potential targets on mRNA level of the TCGA ovarian serous cystadenocarcinoma dataset.

Potential target	cor. coef	p-value	Number of predicted miRNA binding sites
SLC25A3	−0.189	0.000	0
IST1	−0.188	0.000	0
IP6K1	−0.187	0.000	1
CGGBP1	−0.163	0.002	1
ZFC3H1	−0.162	0.002	0
CHTF8	−0.158	0.002	0
EIF4A2	−0.157	0.002	1
ZNF653	−0.150	0.004	2
FANCG	−0.148	0.004	0
DAP3	−0.146	0.005	0
PKNOX1	−0.136	0.009	0
ILK	−0.135	0.009	2
NXF1	−0.134	0.010	0
FAM63A	−0.133	0.010	1
UBA7	−0.129	0.013	0
UIMC1	−0.128	0.014	0
RIC3	−0.124	0.017	0
HMG20A	−0.124	0.017	0
PABPC1L	−0.122	0.019	0
MUTYH	−0.122	0.019	0
USP19	−0.119	0.022	0
BTBD6	−0.118	0.023	0
SYP	−0.114	0.028	0
ESRRA	−0.112	0.032	0
HNRNPC	−0.109	0.036	1
STK33	−0.109	0.036	0
RNF13	−0.106	0.041	0
NFIX	−0.105	0.043	16
RAB8A	−0.103	0.048	1

**Table 2:** miRNA correlation to potential targets on protein level of the TCGA ovarian serous cystadenocarcinoma dataset.

Potential target	cor. coef	p-value	Number of predicted miRNA binding sites
BRD4	−0.290	0.000	0
BAP1C4	−0.205	0.001	0
RBM15	−0.180	0.004	0
PARP1	−0.171	0.006	0
CDH1	−0.153	0.014	0
P16INK4A	−0.148	0.018	2
PRKCD	−0.137	0.028	1
PDCD4	−0.136	0.030	0
EIF4G	−0.135	0.031	0
GATA6	−0.135	0.032	0
SMAD4	−0.134	0.032	0
ADAR1	−0.128	0.040	1
ZIP	−0.127	0.043	0

To evaluate whether the downregulated mRNAs analysed by qRT-PCR are directly targeted by miR-744-5p, the predicted binding sides of all downregulated genes (Table 3) were cloned between the NheI/NotI restriction sites into a pmirGlo Dual Luciferase miRNA target expression vector. By measuring the relative luciferase activity after co-transfection of the expression vector and miR-744-5p, miR-744-5p significantly (p < 0.01) reduced the luciferase activity by binding to HNRNPC-associated 3′UTR binding site (0.76 fold +/− 0.09 fold) and to the binding sites _10, _12 and _16 of NFIX (NFIX_10: 0.74 fold +/− 0.17 fold, NFIX_12: 0.81 fold +/− 0.14 fold and NFIX_16: 0.68 fold +/− 0.11 fold, Fig. 6b,c) when compared to cells transfected with the non-targeting siRNA control (NT). Transfection of miRNA inhibitor anti-miR-744-5p reversed the miR-744-5p mediated inhibitory effect on luciferase activity and was comparable or slightly increased to that observed with the NT control (Fig. 6d

**Table 3:** Predicted binding sides cloned into a pmirGlo Dual Luciferase miRNA target expression vector.

Target mRNA_nr. of binding site	Location in 3′UTR (nt)	Binding sequence	
RAB8A	95–101	3′ acgacaaucgGGaU-CGGGGCGu 5′ 5′ ccucccaccuCCaAcGCCCCGCc 3′	hsa-miR-744 RAB8A
IP6K	2773–2781	3′ acgacaAUCGgGAu---CGGGGCGu 5′ 5′ uacaccUAGCgCUgcccGCCCCGCu 3′	hsa-miR-744 IP6K
ILK_1	77–98	3′ acGACaAuCGGgaucGGGGCGu 5′ 5′ cuCUGgUuGCCu--cCCCCGCc 3′	hsa-miR-744 ILK
ILK_2	226–232	3′ acgACaaUCgggA-UCGGGGCGu 5′ 5′ acaUGggAGggaUcAGCCCCGCc 3′	hsa-miR-744 ILK
HNRNPC	268–276	3′ acgacAAUcggG-AU--CGGGGCGu 5′ 5′ guucaUUAauuCaUAuuGCCCCGCg 3′	hsa-miR-744 HNRNPC
PRKCD	95–102	3′ acGAcaAucggGAuCGGGGCGu 5′ 5′ ugaCUucUgcugCUgGCCCCGCc 3′	hsa-miR-744 PRKCD
ADAR1	866–888	3′ acgacaaucgggaucGGGGCGu 5′ 5′ uccuaggaauauuuuCCCCGCc 3′	hsa-miR-744 ADAR
NFIX_1	185–206	3′ acgacaaucgggaucGGGGCGu 5′ 5′ cuccagcccggggacCCCCGCg 3′	hsa-miR-744 NFIX
NFIX_2	347–368	3′ acgacaaucGGGAucGGGGCGu 5′ 5′ ccuuccuccCCCUgaCCCCGCc 3′	hsa-miR-744 NFIX
NFIX_3	920–941	3′ acgacaaucgggaucGGGGCGu 5′ 5′ cgggccccaggucgcCCCCGCc 3′	hsa-miR-744 NFIX
NFIX_4	1198–1218	3′ acgacaaucgggaucGGGGCGu 5′ 5′ ugccccccgacuuucCCCCGCc 3′	hsa-miR-744 NFIX
NFIX_5	1206–1227	3′ acgacaaucgggaucGGGGCGu 5′ 5′ gacuuucccccgccuCCCCGCc 3′	hsa-miR-744 NFIX
NFIX_6	2511–2517	3′ acgacaauCgGGAuCGGGGCGu 5′ 5′ aagaaccgGgCCU-GCCCCGCc 3′	hsa-miR-744 NFIX
NFIX_7	3058–3064	3′ acgacaauCGGGaucGGGGCGu 5′ 5′ gguccccgGCCCcucCCCCGCa 3′	hsa-miR-744 NFIX
NFIX_8	3358–3365	3′ acgacaaucgggauCGGGGCGu 5′ 5′ cacgcacgcacacgGCCCCGCa 3′	hsa-miR-744 NFIX
NFIX_9	3370–3376	3′ acgacaaucgggaUCGGGGCGu 5′ 5′ cggccccgcacacAGCCCCGCc 3′	hsa-miR-744 NFIX
NFIX_10	3381–3387	3′ acgacaaucgggaucGGGGCGu 5′ 5′ cacagccccgccccaCCCCGCa 3′	hsa-miR-744 NFIX
NFIX_11	3404–3425	3′ acGAcaAUcgGGaucGGGGCGu 5′ 5′ gcCUuaUAcaCCcgcCCCCGCg 3′	hsa-miR-744 NFIX
NFIX_12	3636–3642	3′ acgacaaucGGGaUCGGGGCGu 5′ 5′ cgaggggcaCCCcAGCCCCGCc 3′	hsa-miR-744 NFIX
NFIX_13	3687–3693	3′ acgacaaucGGGAucGGGGCGu 5′ 5′ ggggcgcccCCCUccCCCCGCa 3′	hsa-miR-744 NFIX
NFIX_14	3709–3715	3′ acgacaAuCgGGaUCGGGGCGu 5′ 5′ ccagccUgGgCC-AGCCCCGCu 3′	hsa-miR-744 NFIX
NFIX_15	4058–4064	3′ acGACAAucGgGA-uCGGGGCGu 5′ 5′ guCUGUUccCuCUccGCCCCGCc 3′	hsa-miR-744 NFIX
NFIX_16	4068–4074	3′ acgacaauCGGGauCGGGGCGu 5′ 5′ uccgccccGCCC-cGCCCCGCc 3′	hsa-miR-744 NFIX

). This finding strongly indicates a direct interaction between miR-744-5p and the target sequence of the mRNA. However, predicted binding sides _1, _3, _4, _7, _8, _9, _14 and _15 of NFIX did not modulate luciferase activity, i.e. the same activity was measured when cells were transfected with either miR-744-5p or NT control (data no shown).

In order to assess the effect of miR-744-5p on protein expression, Western Blot analysis of the regulated targets NFIX and HNRNPC were performed. MiR-744-5p transfection caused a substantial reduction in protein expression of NFIX and HNRNPC supporting the luciferase reporter data being direct targets of this miRNA (Fig. 6e, densitometry as Supplementary Fig. S2). As the luciferase activity for the binding sides _10, _12 and _16 of NFIX was significantly (p < 0.01) downregulated, indicating a direct interaction (Fig. 6c, left panel), the NFIX protein expression was profoundly diminished (Fig. 6e, densitometry as Supplementary Fig. S2). Further, PRKCD, ADAR1 and IP6K1 seemed not directly to interact with miR-744-5p (Fig. 6b).

### MiR-744 modulated signalling pathways leading to apoptosis

**Figure 7:**
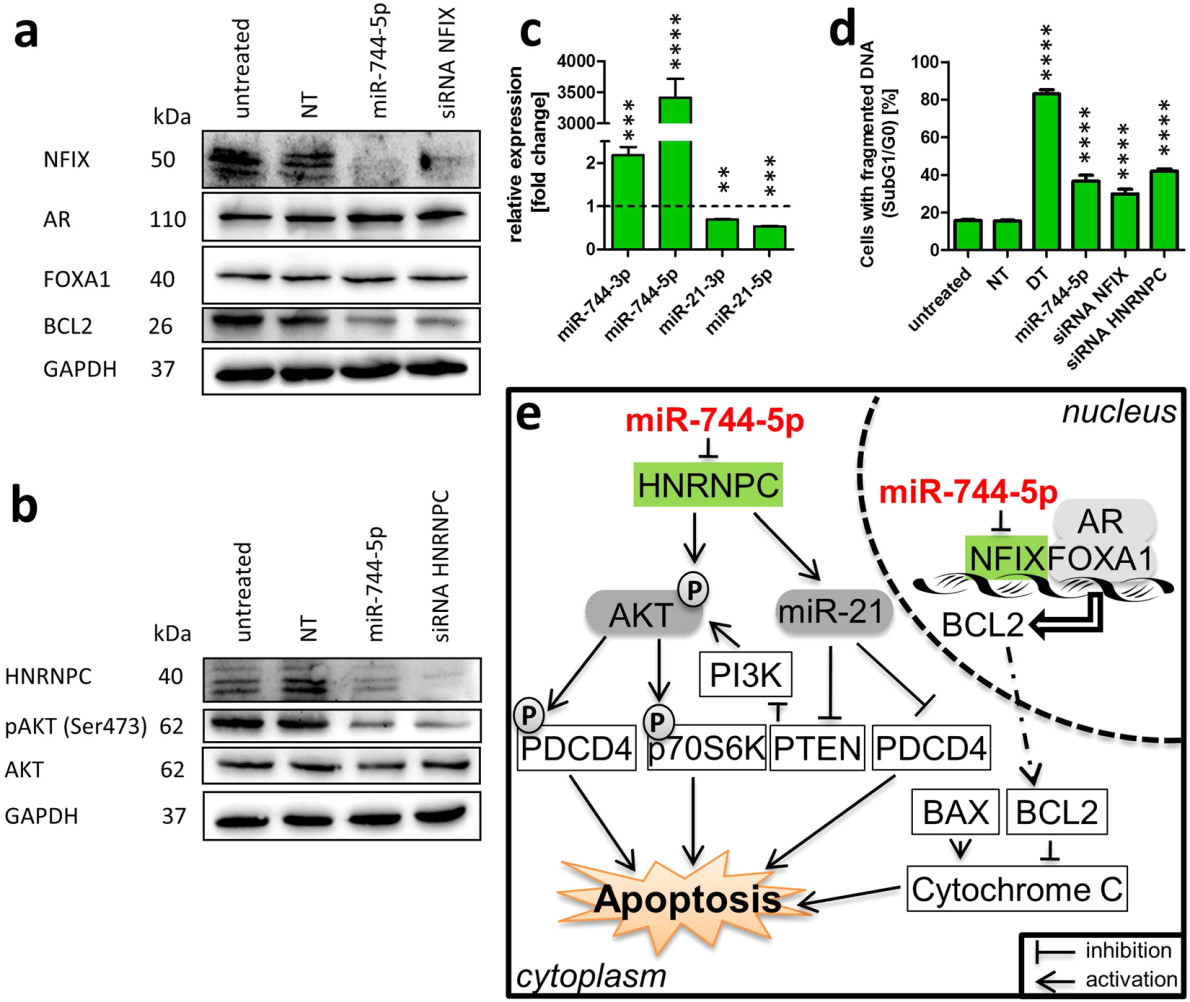
Signalling pathways leading to apoptosis. Western Blot data based on literature research^36,37^ after transfection of SKOV3 cells with miRNA-744 or with siRNA against NFIX (**a**) or HNRNPC (**b**) and qRT-PCR (**c**) confirmed the potential signalling pathway by reduced expression of NFIX and Bcl2 as well as diminished phosphorylation of AKT. By binding to NFIX and HNRNPC miRNA-744 is interfering with the intrinsic apoptotic pathway. Downregulation of HNRNPC resulted in enhanced expression and activation of PDCD4 and p70S6K^36^ (**e**). Reduced NFIX expression, induced by binding of miR-744, leads to reduced expression of Bcl2 leading to apoptosis (**d**). The expression analysis of miR-744 and miR-21 was normalized to the CT value of U6 snRNA^55^. Statistical differences were tested using paired t-test. In order to analyse DNA fragmentation (cells in sub G0/G1, **d**) SKOV3 cells were seeded and transfected as described in Fig. 3 with miR-744 mimics, non-targeting siRNA (NT, negative control for cell death), or siRNA against NFIX or HNRNPC. Statistical analysis was performed by one-way ANOVA followed by Bonferroni post-test (**d**). [n = 3 technical replicates; mean ± SD; ^**^p < 0.01; ^***^p < 0.001; ^****^p < 0.0001]. The cells were harvested for Western Blot analysis 60 h after treatment (a and b). GAPDH was used as loading control.

To elucidate signalling pathways induced by miR-744-5p leading to programmed cell death we analysed putative pathways downstream of miR-744 on protein level based on literature research. Based on Park *et al*.^36^ and Grabowska *et al*.^37^ the protein expression of NFIX, Forkhead Box A1 (FoxA1) and Bcl2 as well as the phosphorylation of AKT Serine/Threonine Kinase (AKT) were analysed by Western blotting 60 h after NT, miR-744-5p or siRNA transfection. As shown in Fig. 7a (densitometry as Supplementary Fig. S3), the expression of Androgen Receptor (AR) and FoxA1 remained unchanged whereas pro-survival Bcl2 decreased after miRNA and siRNA transfection against NFIX. After transfection of miR-744 or of anti-HNRNPC siRNA, the phosphorylation of AKT was reduced when compared to untreated cells or NT control (Fig. 7b). Interestingly, a report by Park *et al*. describes an interaction of HNRNPC with miR-21^36^. We therefore transfected SKOV3 cells with miR-744 and analysed the expression of miR-21 which was reduced in both strands (0.53 fold +/− 0.01 fold for -5p and 0.69 fold +/− 0.01 fold for -3p; Fig. 7c). Flow cytometry analysis 72 h after transfection showed, that downregulation of HNRNPC as well as of NFIX lead to apoptosis. Compared to NT transfected cells (15.48% +/− 0.41%), 29.90% +/− 2.51% of the SKOV3 cells transfected with siRNA against NFIX and 42.03% +/− 1.12% of the anti-HNRNPC siRNA transfected cells showed fragmented DNA (Fig. 7d). Figure 7e summarizes the analysed apoptotic signalling pathways modulated by miR-744-5p via direct binding to NFIX and HNRNPC.

## Discussion

MiRNAs are highly interesting candidate molecules for the generation of novel anticancer therapeutics due to their involvement in the regulation of fundamental cellular processes such as apoptosis. Based on previously performed screenings several pro-apoptotic miRNAs were identified^18^. MiR-744-5p was found to induce apoptosis in various ovarian CA cell lines. In this study, we further validated the pro-apoptotic effect of miR-744-5p in SKOV3 cells and searched for target genes leading to programmed cell death.

Depending on the tissue, miR-744 is reported to have pro-^38^ as well as anti-apoptotic effects^39^. We observed that in old patients (older 67.5 years), registered in the TCGA data bank, high miR-744 expression seemed to be associated with a longer median DFS. Further, Guan *et al*.^39^ and Miyamae *et al*.^12^ described a longer median survival in prostate cancer patients with increased miR-744 levels compared to healthy controls. In addition to these findings, we also observed a reduced miR-744 expression in ovarian CA cell lines SKOV3 and OVCAR3 and an increase in miR-744 expression after treatment with Carboplatin. In line with these observations, Chen and Liu reported a reduced expression of miR-744 in cervical cancer tissue and cell lines^38^. In the current study, we show pro-apoptotic effects of miR-744 in the ovarian CA SKOV3 cells as well as a potential pro-survival function in patients with ovarian serous cystadenocarcinoma.

Ovarian CA is one form of cancer with the highest death-to-incidence ratio^14^. The prognosis is even worse for women who do not respond to available therapies such as Carboplatin due to platinum-resistant tumour cells^40^. A therapy combining Carboplatin and miR-744 might sensitize these cells suggested by the at least additive effects of miR-744 on caspase 3 and −7 activity in SKOV3 cells. Even in Cisplatin resistant A2780 cells, a combined treatment with Carboplatin and miR-744 led to apoptosis.

As the tumour suppressor p53 is frequently mutated or deleted in high-grade ovarian cancer cells^41^ we analysed different ovarian cancer cell lines. The p53 state of the investigated ovarian cancer cell lines differs with the genetic background harbouring either wildtype (A2780), functionally mutated p53 (OVCAR3^p53R248Q^, A2780-cis^p53K351N^) or even lacking p53 expression (SKOV3^p53null^). The finding that in all four cell lines miR-744 induced pro-apoptotic effects, especially in the combination with Carboplatin, might suggest a potential p53-independent mode of miR-744-5p action. This might offer the opportunity to overcome p53 dependent treatment resistance.

However, only little is known about miR-744-5p regulated target genes which are involved in signalling pathways leading to apoptosis.

The heterogeneous nuclear ribonucleoprotein C (HNRNPC) and the nuclear factor I X (NFIX) act as direct target mRNAs of miR-744 involved in apoptotic signalling pathways. Although only a subset of the 16 putative binding sides of NFIX showed downregulated luciferase activity after transfection of miR-744 compared to NT control (Fig. 6c), the protein expression level was clearly reduced indicating an interaction between the miRNA and the target mRNA as described by Kuhn *et al*.^42^.

Based on literature research combined with our results derived from Western Blotting and qRT-PCR we assessed the role of HNRNPC and NFIX in the potential apoptosis signalling pathway. HNRNPC is a ubiquitously expressed RNA-binding protein that shuttles between the nucleus and the cytoplasm and consists of two isoforms with an identical 3′UTR in the mRNA sequence. It is described to influence mRNA metabolism such as pre-mRNA processing and splicing, mRNA transport, stabilization and translation^36^. Park *et al*. reported that silencing HNRNPC in T98G cells leads to apoptosis due to reduced phosphorylation of the serine/threonine kinase AKT2 and the downstream ribosomal serine/threonine kinase p70S6K. Phosphorylated (activated) AKT on Ser473 leads to the expression of pro-survival genes whereas the downstream activated p70S kinase augments protein synthesis and cell proliferation. Programmed cell death 4 (PDCD4) is described as a target of AKT^43^. AKT phosphorylates PDCD4 and thus inhibits its tumour suppressor function (Fig. 7e). Silencing HNRNPC leads to reduced phosphorylation of AKT at Ser473 resulting in diminished PDCD4 phosphorylation and suppressed tumour function^36^. In addition, silencing HNRNPC inhibits the expression of miR-21 which exerts strong pro-survival effects and therefore plays an important role in tumour progression. Reduced miR-21 expression results in increased expression of PDCD4 and reduced expression of phosphatase and tensin homolog (PTEN), both targets of miR-21^44,45^, effects which also would be expected via miR-744 mediated downregulation of miR-21 PTEN dephosphorylates phosphatidylinositol (3,4,5)-trisphosphate (PIP_3_) resulting in the inactivation of the AKT signalling pathway^46^.

The nuclear factor I/X is part of the NFI family consisting of NFIA, NFIB, NFIC and NFIX. They are all defined by their unique DNA-binding domain. NFIX functions as transcriptional regulator of gene expression especially in embryonic development and cell differentiation^43^. Deficiency of NFIX in murine hematopoietic stem and progenitor cells leads to increased apoptosis. A recent report by Grabowska *et al*. describes an interaction between NFIX and the Forkhead box protein A1 (FoxA1) by Förster resonance energy transfer (FRET)^37^. FoxA1 belongs to the transcription family FoxA that includes FoxA2 and FoxA3. They are involved in the regulation of various diseases and programmed cell death^47^. FoxA1 is reported to mediate apoptosis by binding to the Bcl2 promoter and inhibiting the expression of anti-apoptotic Bcl2 in A549 type II pneumocytes^48^. Our data showed an unchanged FoxA1 expression after miR-744-5p mediated NFIX reduction resulting in reduced Bcl2 expression in ovarian CA cells and supports the finding observed in pneumocytes^48^. The reduced expression of Bcl2 will lead to the release of cytochrome C, a component of the intrinsic apoptotic pathway (Fig. 7e)^2,49^ and the detected loss of mitochondrial membrane potential ΔΨm after transfection of miR-744 (Fig. 3b).

Our study shows for the first time a reduction of HNRNPC and NFIX expression on mRNA and protein level induced by the microRNA miR-744 resulting in programmed cell death.

## Experimental Procedures

### Cell culture

T98G (CRL-1690, LGC Standards, Wesel, Germany) and OVCAR3 (HTB-161, LGC Standards) cells were cultured in RPMI-1640 medium (Th Geyer, Renningen, Germany). HOSE 2170^24^, SGBS^19^ and SKOV3 (HTB-77, LGC Standards) cells were cultured in DMEM high glucose medium (Th Geyer). The media were supplemented with 10% (v/v) FBS (Sigma-Aldrich, München, Germany). The A2780 and A2780-cis cells were cultured in RPMI-1640 media supplemented with 20% FBS. All cells were grown at 37 °C and 5% CO_2_. The phenotype of all cell lines was frequently proofed by microscopy. The cell density of the adherent cell culture was measured with the automated single well microscope NyONE (SynenTec Bio Services, Münster, Germany) at a magnification of 10x.

### Transfection with miRNA mimics

Cells were seeded 24 h before transfection in 96-well cell culture plates (Greiner Bio-One). They were transfected with a final concentration of 50 nM (for Fig. 1) or 62.5 nM miRNA mimic (Qiagen, Hilden, Germany). The Silencer Select siRNA against NFIX (siRNA ID: s9503) and HNRNPC (siRNA ID: s6721, Thermo Fisher Scientific, Darmstadt, Germany) were used for the siRNA knockdown experiments. A non-targeting siRNA (AllStars Neg. control, order number: 1027281, negative control for cell death; NT) as well as a human cell death control siRNA (AllStars Cell Death control, order number: 1027299, positive control for cell death; DT) (Qiagen) served as functional controls. ScreenFect A reagent (ScreenFect, Eggenstein-Leopoldshafen, Germany) was mixed 1:125 with dilution buffer. A 1 + 1 mix of ScreenFect A and miRNA mimic was incubated for 20 min at room temperature and put on the cells together with 90 µl of fresh medium^50^. As controls, the cells were treated with 25 µM Etoposide or 0.25 µM Paclitaxel. For combination treatment with miRNAs 55 µM Carboplatin was used (all reagents from Enzo Life Sciences, Lörrach, Germany). The cells were analysed 24 h, 48 h and 72 h after transfection.

### Analysis of apoptotic cells

Apoptotic cells were identified by flow cytometry measuring the amount of cells with reduced DNA content (sub G0/G1) as previously described by Rudner *et al*.^51^. The detached cells were mixed with fresh serum-containing medium and centrifuged for 3 min at 130 × g. The cell pellet was resuspended with a phosphate buffered saline (PBS) buffer containing 0.1% (w/v) Trisodium citrate, 0.05% (v/v) Triton X-100, 10 µg/ml PI and 3.3 µg/ml RNase A (Life Technologies, Darmstadt, Germany). Cells were incubated for 30 min at room temperature in the dark.

About 60,000 cells per transfection were quantified by the MACSQuant Analyser (Miltenyi Biotec, Bergisch-Glattbach, Germany). The detection was performed with the fluorescence channel B2 (585/40 nm filter) and B3 (655–730 nm filter) for determining the subG0/G1 fraction.

For detection of the mitochondrial potential of the cells, a TMRE assay was applied. The cells were detached and mixed with TMRE in culture medium without FBS at a final concentration of 300 nM. 20 min after incubation at 37 °C, the cells were analysed by flow cytometry using the fluorescence channel B2 (585/40 nm filter). 5 µM CCCP (Sigma-Aldrich) was added as a positive control.

The activity of the caspase 3 and -7 was analysed with the CellEvent Caspase-3/7 Green Flow Cytometry Assay Kit (Thermo Fisher Scientific) according to the manufacturer’s protocol and measured with the fluorescence channels B1 (525/50 nm) and B3 (655–730 nm filter).

### Apoptosis assay with long-term video-microscopy

The activation of caspases was measured by long-term video-microscopy. The cells were transfected as described above and/or treated with Carboplatin. 1 h after treatment IncuCyte Caspase-3/7 Green Apoptosis Assay Reagent or IncuCyte AnnexinV Red Reagent (Sartorius, Goettingen, Germany) was added to a final concentration of 5 nM as well as PI at a final concentration of 10 μg/ml (Sigma Aldrich)^52^. The cells were placed into the IncuCyte ZOOM Live-Cell Analysis System detecting red and green florescence. The imaging system is placed in an incubator with standard culture conditions. Pictures from each well in two different positions were taken automatically every hour with a 20x objective and analysed with the IncuCyte ZOOM Software for the amount of fluorescent stained cells. The data were visualized using GraphPad Prism 5 software.

### RT-PCR

The extraction of total RNA was done using the miRNeasy Mini Kit (Qiagen). 1000 ng of isolated RNA was transcribed with the miScript II RT Kit (Qiagen) using 5x miScript HiSpec Buffer and an incubation time of 60 min at 37 °C. The 1:30 diluted cDNA was further analysed in the Roche Light Cycler 480 using GreenMasterMix (Genaxxon Bioscience, Ulm, Germany). To analyse the expression of potential target genes, 1000 ng of isolated RNA were transcribed using the Transcriptor High Fidelity cDNA Synthesis Kit from Roche (Penzberg, Germany). For miRNA detection the following primers were used:

hsa-miR-744-3p (5′-CTGTTGCCACTAACCTCAACCT-3′), hsa-miR-744-5p (5′-TGCGGGGCTAGGGCTAACAGCA-3′), hsa-miR-21-3p (5′-CAACACCAGTCGATGGGCTGT-3′) or hsa-miR-21-5p (5′-TAGCTTATCAGACTGATGTTGA-3′) were used together with the universal reverse primer from the miScript PCR Starter Kit (Qiagen). U6 snRNA primer forward (5′-CTCGCTTCGGCAGCACA-3′) and U6 snRNA primer reverse (5′-AACGCTTCACGAATTTGCGT-3′) were used as housekeeping control. For target identification the following primers were used: ADAR1-FW (5′-TTCGAGAATCCCAAACAAGG-3′), ADAR1-RV (5′-CTGGATTCCACAGGGATTGT-3′), HNRNPC-FW (5′-AGAACCCGGGAGTAGGAGAC-3′), HNRNPC-RV (5′-TCTCACAAAGCCGAAAACAA-3′), ILK-FW (5′-GAGTTCCCCGGAGAAGGAT-3′), ILK-RV (5′-GAGGACTGTGGAGTGATCCAG-3′), IP6K1-FW (5′-CCTGTCCCTTGAGACCTCTG-3′), IP6K1-RV (5′-GGACCTCTGAGTGGCTGTG-3′), MAP2K4-FW (5′-TGTTTGCTTCTTGCCATCAC-3′), MAP2K4-RV(5′-CAGGGGAAGGGAAAGAGATT-3′), NFIX-FW (5′-ACTCCCCGTACTGCCTCAC-3′), NFIX-RV (5′-TGCAGGTTGAACCAGGTGTA-3′), PPIA-FW (5′-ATGCTGGACCCAACACAAAT-3′), PPIA-RV (5′-TCTTTCACTTTGCCAAACACC-3′), PRKCD-FW (5′-ATTATCCCCGCTGGATCAC-3′), PRKCD-RV (5′-CTTGGTTGGTTCCCTTTCAA-3′), RAB8A-FW (5′-GCCCTCGACTATGGAATCAA-3′) and RAB8A-RV (5′-TGGCGAGAGTGAAAAATGC-3′).

### Western Blotting

For protein expression analysis the cells were lysed with radioimmunoprecipitation assay (RIPA) buffer containing 1% (v/v) Nonidet P40, 0.5% (w/v) Sodium-Deoxycholate, 1 mM EDTA and 0.5 µM DTT. The protein concentration was measured with a Bicinchoninic acid assay detecting the OD of the solution at a wave length of 562 nm. 35 µg protein were loaded on a 8% or 12.5% gel and run for 1.5 h. The proteins were transferred onto a PVDF-membrane (Carl Roth, Karlsruhe, Germany). The membrane was blocked with a 5% (w/v) BSA-solution in TBST for 1 h. The incubation of the primary antibody followed overnight at 4 °C. The antibodies against PARP (cs#9542), cleaved PARP (cs#9541), Caspase 3 (cs#9662) and cleaved Caspase 3 (cs#9664) obtained from Cell Signaling Technology (Danvers, United States) were diluted 1:1,000 in blocking solution.

For detection of target proteins the following antibodies were used:

HNRNPC (1:1,000; #91327; Cell Signaling Technology), NFIX (1:250; HPA007533; Atlas Antibodies AB, Bromma, Sweden), Bcl2 (1:500; sc-509; Santa Cruz, Heidelberg, Germany), HNF-3α (FoxA1; 1:250; sc-514695; Santa Cruz Biotechnology), AR (1:250; sc-7305; Santa Cruz Biotechnology), Akt1/2/3 (1:500; sc-81434; Santa Cruz Biotechnology), p-Akt1/2/3 Ser473 (1:500; sc-514032; Santa Cruz Biotechnology) and GAPDH (1:5,000; MA5-15738; Thermo Fisher Scientific).

The secondary anti-rabbit IgG, HRP-linked (cs#7074, Cell Signaling Technology) or anti-mouse IgG, HRP linked (A4416, Sigma Aldrich) were diluted 1:10,000 in blocking solution and incubated for 1 h at room temperature. The chemiluminescence signal was detected with the Fusion FX (Vilber Lourmat, Eberhardzell, Germany) using the appropriate software and the Immobilon Western Chemiluminescent HRP substrate (Thermo Fisher Scientific).

### TCGA analysis

To examine the correlation between the miR-744 expression and potential targets, level 3 miRNA, mRNA and protein expression data of the “Ovarian serous cystadenocarcinoma” data set were downloaded from the cancer genome atlas (TCGA) data base (www.tcga-data.nci.nih.gov/tcga) using the “firehose-get“ command-line tool (https://confluence.broadinstitute.org/display/GDAC/Download). A detailed description of the clinical characteristics of the cohort can be found in a study by Cancer Genome Atlas Research Network^53^.

For the delineation of the prognostic effect of miR-744 in different age groups, the dataset defined by the first quartile, mean, median and the third quartile of the age distribution (range: 26–89 years, mean: 67.5 years), differential survival analysis for patients with high versus low expression of miR-744 binding partners, using mean expression as threshold and DFS as endpoint, was conducted. For this, the log-rank test was applied on the resulting cox-proportional hazard-models and for the purpose of visualization Kaplan-Meier-Plots were generated.

For the identification of potential targets the miRNA expression data were negatively correlated to the mRNA and protein expression data from each patient. The online gene classification software PANTHER (Protein Analysis Through Evolutionary Relationships)^33^ was used to cluster the potential targets for apoptotic functions. From these, potential targets with miR-744-5p binding sites referring to the database microRNA.org^34^ and TargetScanHuman^35^ were selected.

### Luciferase reporter assay

Complementary oligonucleotide pairs comprising a portion of putative miRNA binding sites were synthesized (Thermo Fisher Scientific), annealed and cloned into the pmirGlo Dual Luciferase miRNA target expression vector (Promega Corporation, USA) between the NheI/NotI restriction sites of the multiple cloning site downstream of a luciferase gene.

For luciferase assays, HEK293T cells were seeded into 96-well cell culture pates. 24 h later, the cells were co-transfected with 200 ng of the pmirGlo Dual Luciferase miRNA target expression vector and miR-744-5p, microRNA inhibitor anti-miR-744-5p or non-targeting siRNA control (NT) at a final concentration of 50 nM using Lipofectamine 3000 (Thermo Fisher Scientific). Three days after transfection, cells were lysed with the Dual-Glo Reagent (Dual-Glo Luciferase Assay System; Promega Corporation, Mannheim, Germany) and luciferase activity was quantified on a SpectraMax M5e microplate reader (Molecular Devices, Sunnyvale, USA). After calculating the ratio of firefly luminescence to the luminescence from Renilla, the experimental well ratio was normalized to the ratio of the control wells.

### Statistical analysis

Data in general were expressed as mean +/− SD. Statistical analysis was carried out using GraphPad Prism Version 5.04. The corresponding statistical test and the level of significance are indicated in each figure legend. For TCGA analysis all calculations were conducted using the R statistical platform^54^ using functions from the CRAN package survival (www.cran.r-project.org/web/packages/survival).

## Electronic supplementary material

Supplementary Dataset 1
