# Supplementary material for: MiR-744-5p inducing cell death by directly targeting HNRNPC and NFIX in ovarian cancer cells
Source: Sci Rep. 2018 Jun 13;8:9020. doi: 10.1038/s41598-018-27438-6 (PMC5998049; doi:10.1038/s41598-018-27438-6)
Supplement: Supplementary file 1 — Supplementary Dataset 1 [file 41598_2018_27438_MOESM1_ESM.docx]

**Supplementary data to**

**MiR-744-5p inducing cell death by directly targeting HNRNPC and NFIX in ovarian cancer cells**

Michael Kleemann^1,2,^*, Helga Schneider^1^, Kristian Unger^3^, Philip Sander^4^, E. Marion Schneider^4^, Pamela Fischer-Posovszky^5^, René Handrick^1^, Kerstin Otte^1^

^1^ Institute of Applied Biotechnology, University of Applied Sciences Biberach, Hubertus-Liebrecht-Str. 35, 88400 Biberach, Germany

^2^ University of Ulm, Faculty of Medicine, Albert-Einstein-Allee 11, 89079 Ulm, Germany

^3^ Research Unit Radiation Cytogenetics, Helmholtz Center Munich, German Research Center for Environmental Health, Ingolstädter Landstr. 1, 85764 Neuherberg, Germany

^4^ University Medical Center Ulm, Division of Experimental Anesthesiology, Albert-Einstein-Allee 23, 89081 Ulm, Germany

^5^ University Medical Center Ulm, Division of Pediatric Endocrinology and Diabetes, Department of Pediatrics and Adolescent Medicine, Eythstr. 24, 89075 Ulm, Germany

**Supplementary Figure S1**

**Unprocessed Western Blot supporting Figure 3: Time course and hallmarks of miR-744 induced apoptosis**

Parp GAPDH

kDa

135

100

75

kDa

63

48

35

Caspase 3 / cleaved Caspase 3

kDa

63

48

35

25

20

17

**Densitometrical analysis of Western Blots for Figure 3**

**Supplementary Figure S2**

**Unprocessed Western Blot supporting Figure 6: HNRNPC and NFIX as direct targets for miR-744**

NFIX HNRNPC

kDa

63

48

35

kDa

63

48

35

kDa

75

63

48

35

kDa

75

63

48

35

GAPDH

kDa

75

63

48

35

kDa

75

63

48

35

**Densitometrical analysis of Western Blots for Figure 6**

**Supplementary Figure S3**

**Unprocessed Western Blot supporting Figure 7: Signalling pathways leading to apoptosis**

NFIX AR

kDa

63

48

35




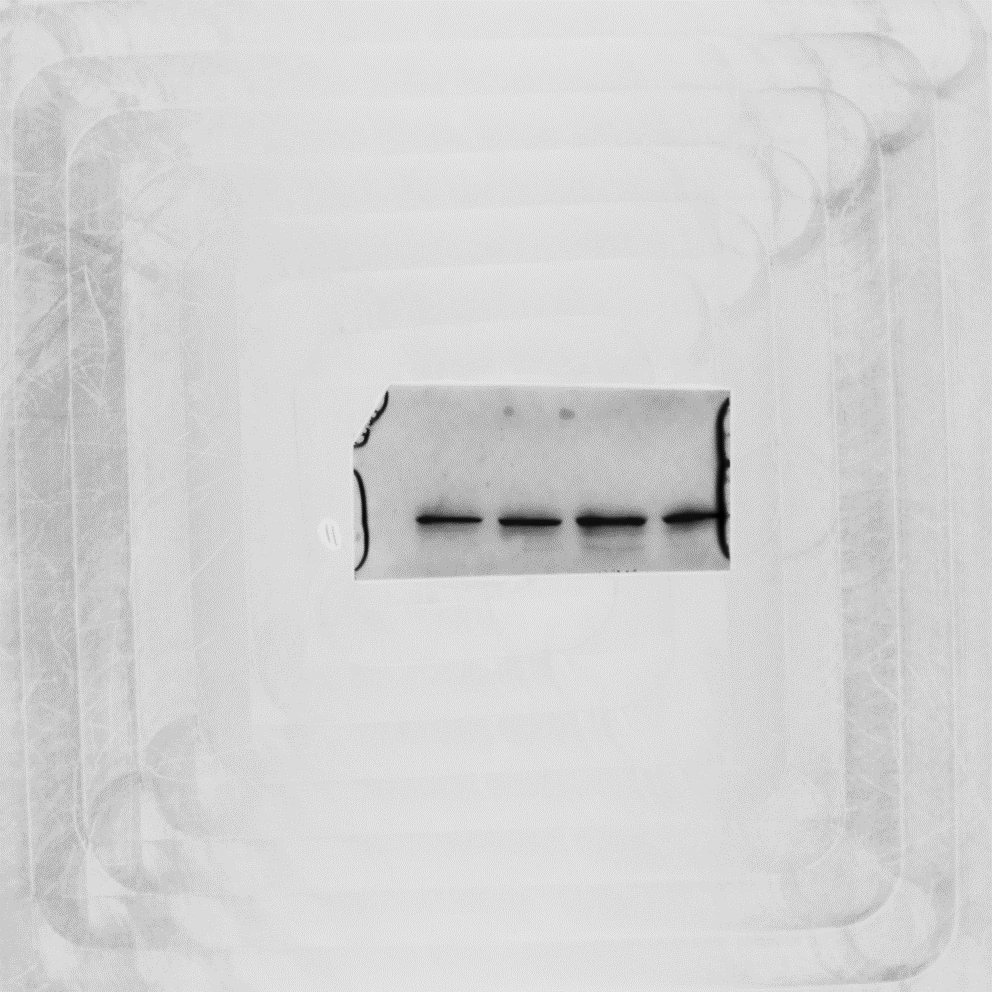


kDa

135

100

75

FOXA1 BCL2

kDa

25

20

17

kDa

48

35

25


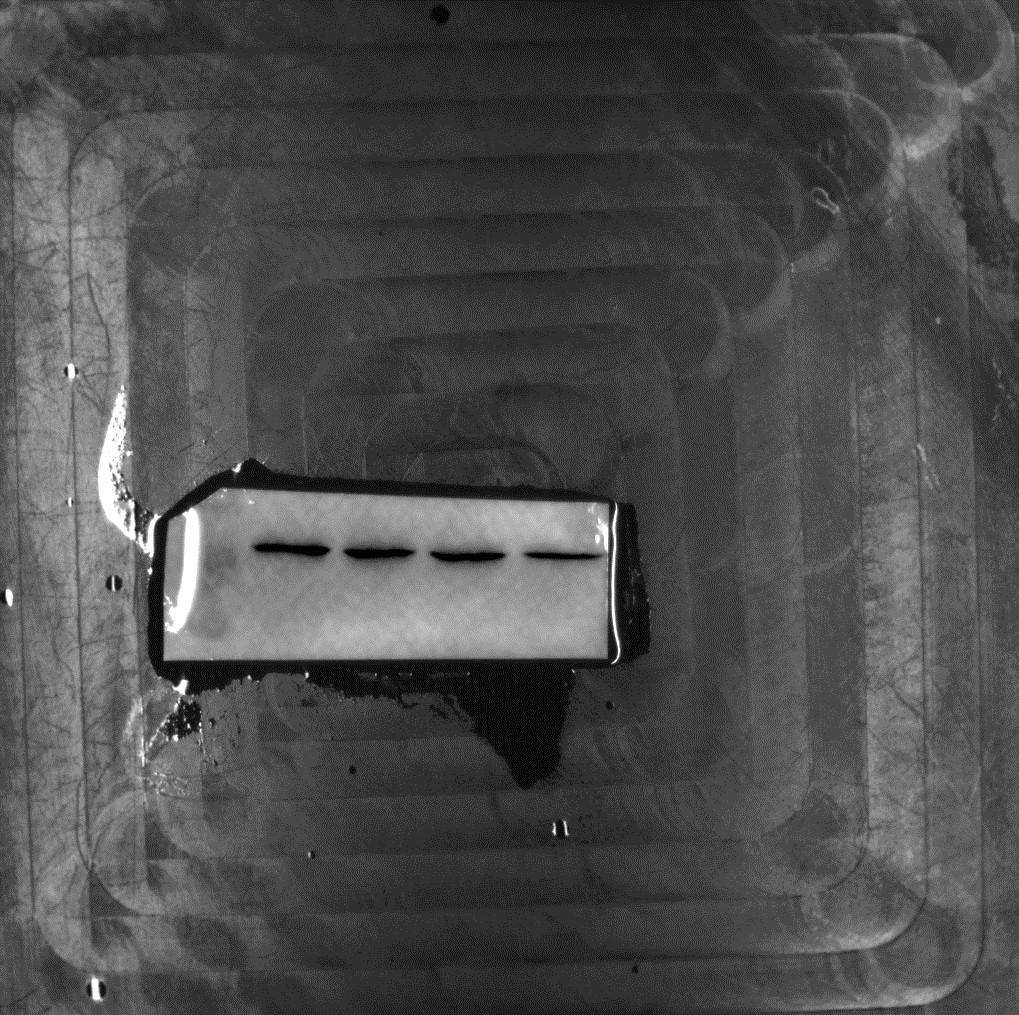

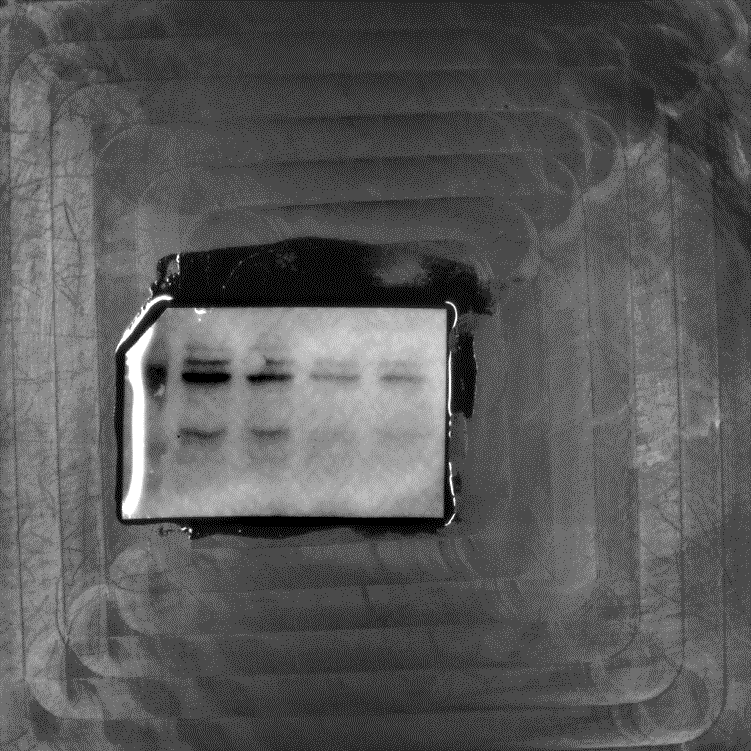


GAPDH

kDa

63

48

35





HNRNPC GAPDH







kDa

48

35

kDa

48

35

pAKT AKT

kDa

75

63


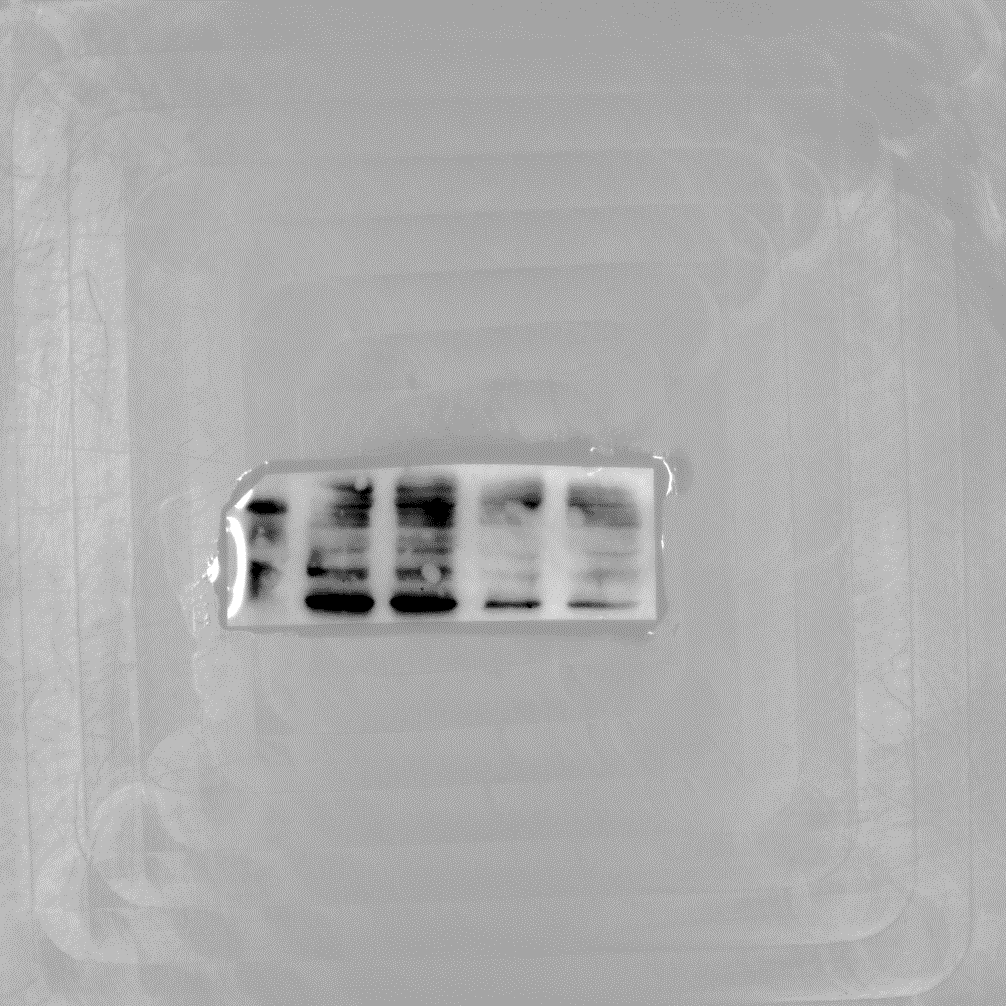


kDa

75

63

**Densitometrical analysis of Western Blots for Figure 7**
